# Differential Accumulation of Aroma Compounds in Normal Green and Albino-Induced Yellow Tea (*Camellia sinensis*) Leaves

**DOI:** 10.3390/molecules23102677

**Published:** 2018-10-18

**Authors:** Fang Dong, Lanting Zeng, Zhenming Yu, Jianlong Li, Jinchi Tang, Xinguo Su, Ziyin Yang

**Affiliations:** 1 Guangdong Food and Drug Vocational College, Longdongbei Road 321, Tianhe District, Guangzhou 510520, China; dongfangxyz@163.com; 2 Key Laboratory of South China Agricultural Plant Molecular Analysis and Genetic Improvement & Guangdong Provincial Key Laboratory of Applied Botany, South China Botanical Garden, Chinese Academy of Sciences, Xingke Road 723, Tianhe District, Guangzhou 510650, China; zenglanting@scbg.ac.cn (L.Z.); zhenming311@scbg.ac.cn (Z.Y.); 3 College of Advanced Agricultural Sciences, University of Chinese Academy of Sciences, No.19A Yuquan Road, Beijing 100049, China; 4 Tea Research Institute, Guangdong Academy of Agricultural Sciences & Guangdong Provincial Key Laboratory of Tea Plant Resources Innovation and Utilization, Dafeng Road 6, Tianhe District, Guangzhou 510640, China; skylong.41@163.com (J.L.); tangjinchi@126.com (J.T.)

**Keywords:** albino, aroma, *Camellia sinensis*, geranyl diphosphate, light-sensitive, linalool, tea

## Abstract

Tea (*Camellia sinensis*) cultivars with green leaves are the most widely used for making tea. Recently, tea mutants with white or yellow young shoots have attracted increasing interest as raw materials for making “high-quality” tea products. Albino teas are generallycharacterized as having metabolites of relatively high amino acid content and lower catechin content. However, little is known about aroma compounds in albino tea leaves. Herein, we compared original normal leaves (green) and light-sensitive albino leaves (yellow) of cv. Yinghong No. 9. GC-MS was employed to analyze endogenous tea aroma compounds and related precursors. Quantitative real time PCR was used to measure expression levels of genes involved in biosyntheses of tea aromas.The total contents of most endogenous free tea aromas, including aroma fatty acid derivatives, aroma terpenes, and aroma phenylpropanoids/benzenoids, and their glycosidically bound aroma compounds, were lower in yellow leaves than in green leaves. The content of the key precursor geranyl diphosphate (GDP) and expression levels of key synthetic genes involved in the formation of linalool, a major aroma compound in cv. Yinghong No. 9, were investigated. Linalool content was lower in albino-induced yellow leaves, which was due to the lower GDP content compared with normal green leaves.

## 1. Introduction

The drink made from tea (*Camellia sinensis*) leaves is the second most popular beverage worldwide after water. This popularity is attributed to tea leaves having health benefits and containing flavor related metabolites, such as polyphenols, caffeine, amino acids, aroma compounds, vitamins, and carbohydrates [1,2,3]. These metabolites can be affected by preharvest agronomy management and postharvest manufacturing processes [3,4,5]. Furthermore, these metabolites have different distributions in different tea varieties. In general, tea cultivars with green leaves are the most widely used for making tea. In recent years, tea mutants with white or yellow young shoots have attracted increasing interest from tea researchers and tea manufacturers as potential raw materials for making “high-quality” tea products. According to their different responses to the environment, albino teas can be divided into ecologically sensitive types (including light-sensitive and low-temperature-sensitive types), in which leaf colors change to white or yellow under certain environmental conditions, or ecologically sensitive types where the leaf color is permanently white or yellow regardless of the environment [6,7]. Temperature-sensitive mutants grow white or yellow shoots below certain temperatures, such as 20 °C, during early spring, with examples including ‘Xiaoxueya’ and ‘White leaf No. 1’ cultivars [8,9]. Light-sensitive mutants grow yellow shoots under strong light illumination, with examples including ‘Jinguang’, ‘Yu-Jin-Xiang’, and ‘Huangjinya’ cultivars [10,11,12]. These albino tea leaves have white or yellow shoots owing to chlorophyll deficiencies and, in terms of metabolite characteristics, generally have relatively high amino acid contents and lower catechin contents [6,7]. However, little is known about the occurrence of aroma compounds in albino tea leaves. Although no reported reference is available, most tea products made from albino-induced yellow leaves generally showed weak aroma properties according to information from tea manufacturers. From the viewpoint of plants, the albino tea leaves have defects in the chloroplasts [13], which can affect the biosynthesis of specialized metabolites such as catechins, which are lower in content in albino-induced yellow tea leaves [6,7]. Accumulation of proteinaceous amino acids in albino-induced yellow tea leaves possibly results from degradation of chloroplast-located proteins. Higher content of some non-proteinaceous amino acid such as l-theanine in albino tea leaves is due to weak catabolism of the amino acid in albino tea leaves [13]. The objective of this study is to investigate whether albinism affects tea aroma profiles and if it is a positive or negative influence. Aroma is an important factor affecting the character and quality of tea [4,5]. In general, phenolic compounds and amino acids account for 18–36% and 1–4% of tea content, respectively, while aroma compounds account for less than 0.03% [3]. Therefore, improving tea aroma quality is of significant interest and has attracted increasing research attention. Understanding the biosyntheses and regulation mechanisms of tea aroma compounds is key to safely and effectively improving tea quality [5]. In fresh tea leaves with living leaf cells, tea aroma compounds are mainly classified into aroma fatty acid derivatives (VFADs), aroma terpenes (VTs), and aroma phenylpropanoids/benzenoids (VPBs) according to the different biosynthetic pathways, and similar to other plants [4,5]. In fresh tea leaves, not many aroma compounds are detected, and they occur conservatively in most tea cultivars. During the tea manufacturing process, many aroma compounds, which may involve some new compounds, are produced [4,5]. 

In this study, to exclude the effects of genetic background, geography, and climate, *C. sinensis* cv. Yinghong No. 9 original cultivar (green) and its light-sensitive mutant (albino-induced yellow), which shares the same genetic background were sampled in pairs under the same growth conditions. We compared the aroma profiles (VFADs, VTs, and VPBs), major aroma compound related key precursor metabolite levels, and key synthetic gene expressions, of normal green tea leaves and albino-induced yellow tea leaves. This information will advance our understanding of the profiles of tea aromas and their formation in albino tea leaves.

## 2. Results

### 2.1. Comparisons of Endogenous Free Aroma Compound Contents between Albino-Induced Yellow Leaves and Normal Green Leaves

**Figure 1:**
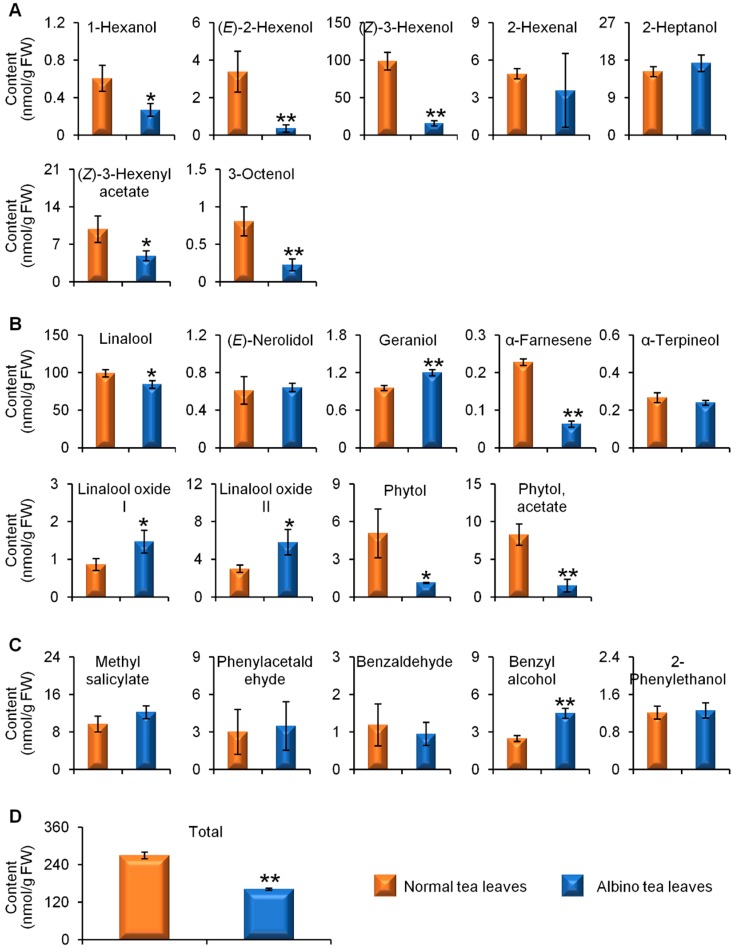
Differential accumulation of endogenous free aroma compounds in normal green and albino-induced yellow tea (*Camellia sinensis*) leaves (cv. Yinghong No. 9) collected in April. (**A**) aroma fatty acid derivatives; (**B**) aroma terpenes; (**C**) aroma phenylpropanoids/benzenoids; (**D**) total content of aroma compounds shown in (**A**–**C**). The identification and quantitative analysis of the aroma compounds is summarized in Table S1 (Supplementary Information). All data are expressed as mean ± S.D. (*n* = 3). Significant differences between normal and albino tea leaves are indicated (* *p* ≤ 0.05, and ** *p* ≤ 0.01). FW, fresh weight.

**Figure 2:**
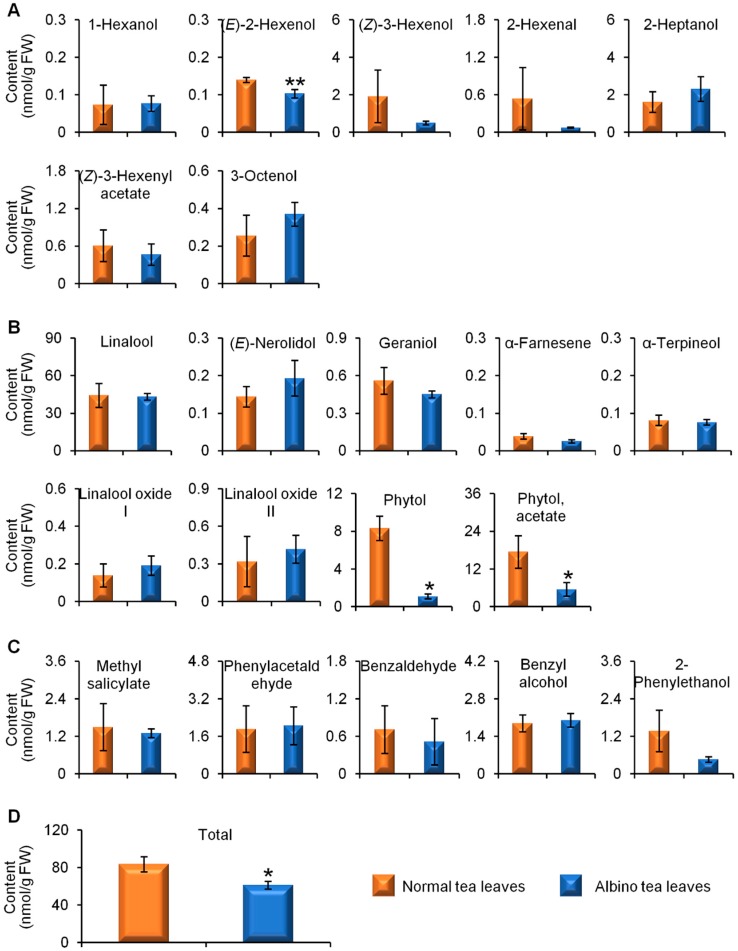
Differential accumulation of aroma compounds in normal green and albino-induced yellow tea (*Camellia sinensis*) leaves (cv. Yinghong No. 9) collected in August. (**A**) aroma fatty acid derivatives; (**B**) aroma terpenes; (**C**) aroma phenylpropanoids/benzenoids; (**D**) total content of aroma compounds shown in (**A**–**C**). The identification and quantitative analysis of the aroma compounds is summarized in Table S1 (Supplementary Information). All data are expressed as mean ± S.D. (*n* = 3). Significant differences between normal and albino tea leaves are indicated (* *p* ≤ 0.05, and ** *p* ≤ 0.01). FW, fresh weight.

**Figure 3:**
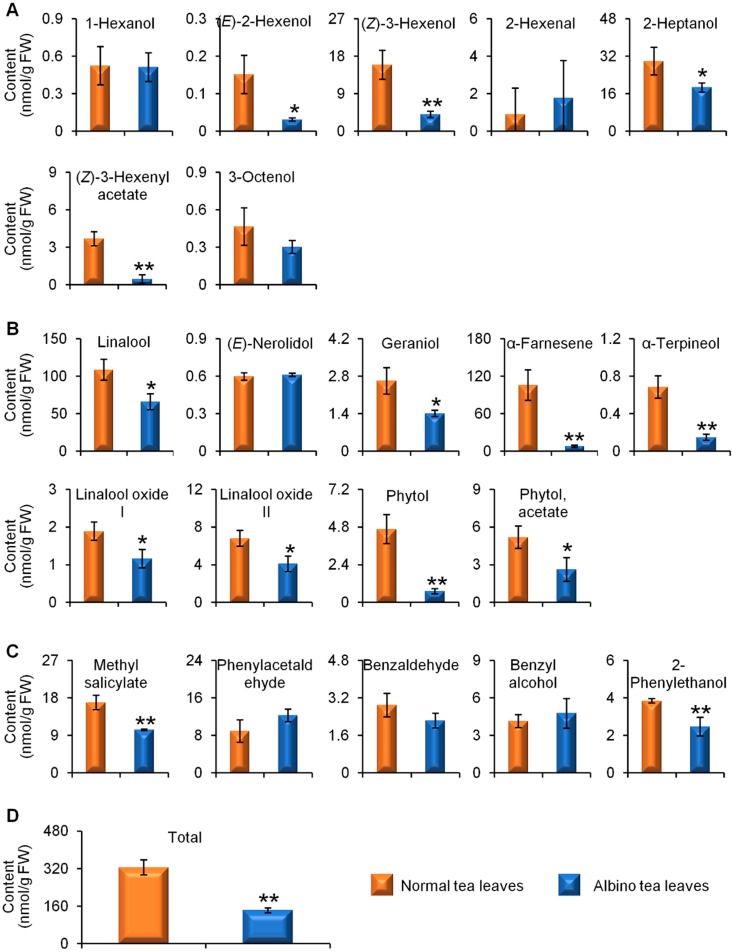
Differential accumulation of aroma compounds in normal green and albino-induced yellow tea (*Camellia sinensis*) leaves (cv. Yinghong No. 9) collected in December. (**A**) aroma fatty acid derivatives; (**B**) aroma terpenes; (**C**) aroma phenylpropanoids/benzenoids; (**D**) total content of aroma compounds shown in (**A**–**C**). The identification and quantitative analysis of the aroma compounds is summarized in Table S1 (Supplementary Information). All data are expressed as mean ± S.D. (*n* = 3). Significant differences between normal and albino tea leaves are indicated (* *p* ≤ 0.05, and ** *p* ≤ 0.01). FW, fresh weight.

Figure S1 (supplementary information) shows GC-MS TIC chromatograms of endogenous free aroma compounds in normal green and albino-induced yellow tea leavescollected in April, August, and December. Both normal green tea leaves and albino-induced yellow leaves contained the three major classes of aromas, namely VFADs (Figure 1A), VTs (Figure 1B), and VPBs (Figure 1C). In the spring tea leaves collected in April, although not all of the content of the aroma compounds were lower in the albino-induced yellow leaves than in the normal green leaves, the content of the two most abundant free aroma compounds, (*Z*)-3-hexenol and linalool (Figure 1A,B), was significantly less, which led to a lower total content of the detected aromas in albino-induced yellow leaves than in normal green leaves (Figure 1D). To investigate whether different seasons affected the endogenous free aroma compounds in albino-induced yellow leaves and normal green leaves, we compared the aroma profiles of both types of tea leaves picked in August (summer-autumn tea) (Figure 2) and December (winter tea) (Figure 3). In the tea samples picked in August and December, the total content of the detected aromas was lower in the albino-induced yellow leaves than in the normal green leaves (Figure 2D and Figure 3D), which showed similar trends to those in the spring tea (Figure 1D). In addition, endogenous free aroma compound contents were affected by tea seasons. The aroma compounds of spring tea and winter tea were generally higher than those of summer-autumn tea (Figure 1, Figure 2 and Figure 3).

### 2.2. Comparison of Glycosidically Bound Aroma Compound Contents and Related Gene Expression Levels between Albino-Induced Yellow Leaves and Normal Green Leaves

**Figure 4:**
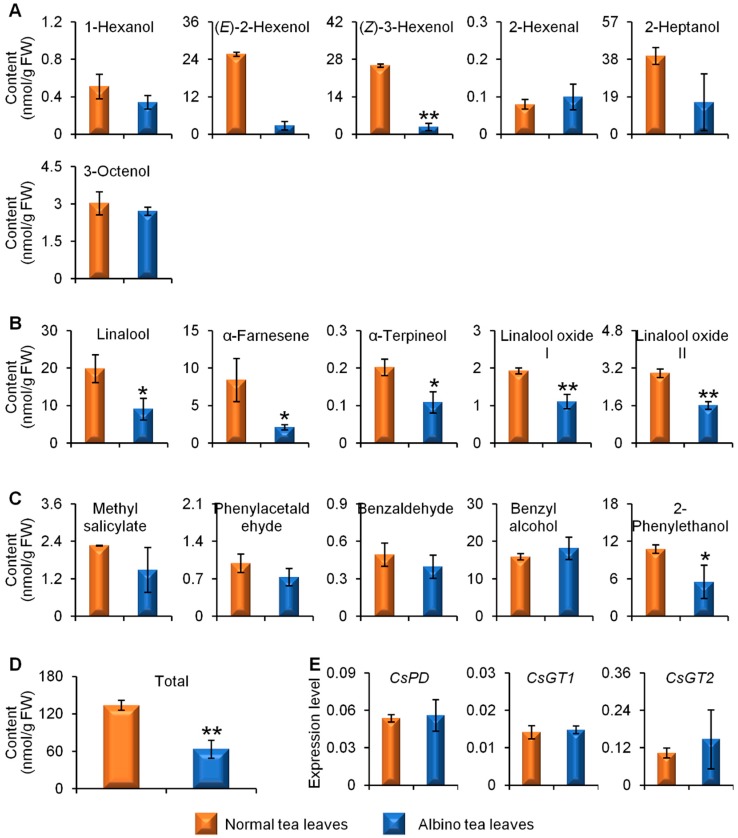
Differential accumulation of glycosidically bound aromas (GBVs)(**A**–**D**) and expression levels of genes involved in aroma compounds synthesis from glycosidic hydrolysis (**E**) in normal green and albino-induced yellow tea (*Camellia sinensis*) leaves (cv. Yinghong No. 9) collected in April. The content of GBVs were calculated based on the enzyme reaction products under β-primeverosidase and β-glucosidase catalyzation. (**A**) aroma fatty acid derivatives; (**B**) aroma terpenes; (**C**) aroma phenylpropanoids/benzenoids; (**D**) total content of aroma compounds shown in (**A**–**C**). The identification and quantitative analysis of the aroma compounds is summarized in Table S1 (Supplementary Information). FW, fresh weight. (**E**) *PD*, β-*primeverosidase*; *GT*, *glycosyltransferase*. All data are expressed as mean ± S.D. (*n* = 3). Significant differences between normal and albino tea leaves are indicated (* *p* ≤ 0.05, and ** *p* ≤ 0.01).

In addition to their free forms, aroma compounds can occur in plants as glycosidically bound forms that are more water soluble and less reactive than their free aglycone counterparts. We also compared glycosidically bound aroma compounds in albino-induced yellow leaves and normal green leaves. The results showed that the content of most glycosidically bound aroma compounds was lower in albino-induced yellow leaves than in normal green leaves (Figure 4A–D). Furthermore, the expression levels of glycosidically conjugated aroma compound formation and hydrolysis-related genes (β-primeverosidase (PD), glycosyltransferases (GT1 and GT2)) showed no significant differences between albino-induced yellow leaves and normal green leaves (Figure 4E).

### 2.3. Comparison of Linalool Synthase Gene Expression Levels and GDP Content between Albino-Induced Yellow Tea Leaves and Normal Green Tea Leaves

**Figure 5:**
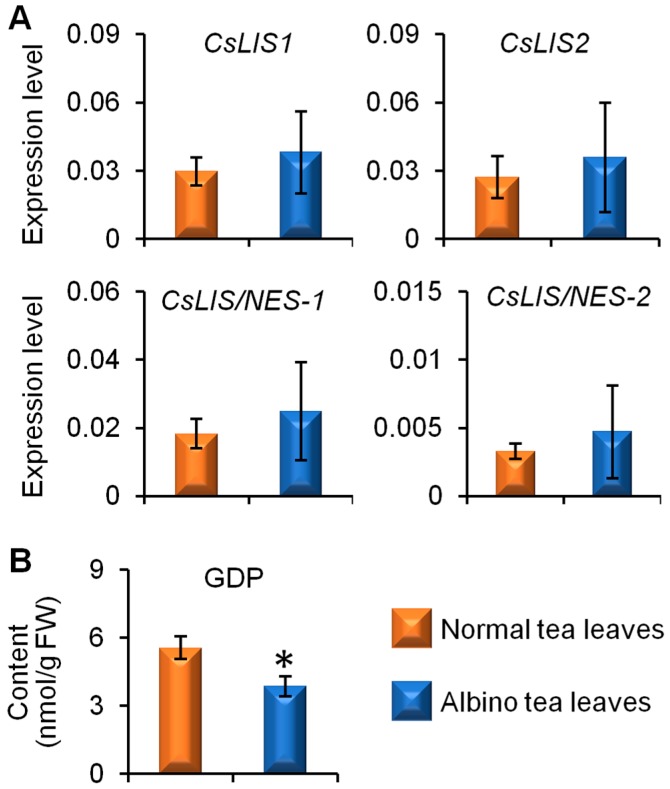
Differential expression levels of genes involved in linalool synthesis (**A**) and accumulation of geranyl diphosphate (GDP) (**B**) in normal green and albino-induced yellow tea (*Camellia sinensis*) leaves (cv. Yinghong No. 9) collected in April. (**A**) *LIS*, *linalool synthase*; *LIS/NES*, *linalool synthase/(E)-nerolidol synthase*. (**B**) FW, fresh weight. All data are expressed as mean ± S.D. (*n* = 3). Significant differences between normal and albino tea leaves are indicated (* *p* ≤ 0.05).

In the present study, linalool is a major endogenous free aroma compound in cv. Yinghong No. 9 tea leaves (Figure 1, Figure 2 and Figure 3). Therefore, we investigated the reason for the lower linalool content in albino-induced yellow tea leaves. The four linalool synthase genes involved in linalool formation from GDP, namely, *CsLIS1*, *CsLIS2*, *CsLIS/NES-1*, and *CsLIS/NES-2*, showed no significant differences between the albino-induced yellow leaves and normal green leaves (Figure 5A). However, the GDP content in albino-induced yellow leaves was lower than that in normal green leaves (Figure 5B), which might be a reason for the lower amount of linalool in albino-induced yellow tea leaves.

## 3. Discussion

Obviously, albino tea leaves are yellow due to a lack of chlorophyll compared with the original green cultivars, which leads to defects in the chloroplast ultrastructure and composition, and a reduction in the number of chloroplasts. In contrast, the original green cultivars have normal chloroplast grana, stroma, thylakoids, and starch grains [8]. The albinism mechanism in albino tea cultivars and changes in non-aroma metabolites during leaf development or shade treatment have been studied using proteomics, transcriptomics, and metabolomics analysis [10,11,12,13,14]. In previous studies, the tea samples selected have different genetic backgrounds, and different geographic or climate characteristics, hence, these factors may interfere with results concerning the effects of albinism on metabolites in tea leaves. In our previous study, *C. sinensis* cv. Yinghong No. 9 original cultivar (green) and its light-sensitive mutant (albino-induced yellow), which shares the same genetic background, were used in pairs under the same growth conditions to investigate the differential accumulation of specialized metabolite l-theanine in green and albino-induced yellow tea leaves [13]. Although the availability of ethylamine (a biosynthetic precursor of l-theanine) is crucial for the differential accumulation of l-theanine between tea and other plants [15], weak l-theanine catabolism was responsible for the higher l-theanine content in albino-induced yellow leaves compared with normal green leaves [13]. The relatively low catechin contents in the albino tea leaves might also be related to the limited catechin biosynthesis in albino tea leaves [11,12]. Complete dark treatment of normal green tea leaves led to etiolated leaves, which had the same appearance as albino-induced yellow leaves [16]. The dark treatment significantly increased the free amino acid content and decreased the soluble protein content in tea leaves. Chloroplast numbers were lower in dark-treated leaves and the soluble protein content in chloroplasts isolated from dark-treated leaves was lower compared with the control, suggesting that proteolysis of the chloroplast proteins contributed to amino acid accumulation in dark-treated leaves [17]. The accumulation of proteinaceous amino acids possibly resulted from the degradation of chloroplast-located proteins. Previous reports have mostly focused on the effects of albinism on non-aroma metabolites in tea leaves. In the present study, the amount of major endogenous free tea aroma compounds (aroma metabolites) was lower in yellow leaves than in green leaves (Figure 1, Figure 2 and Figure 3). Furthermore, the content of most glycosidically bound aroma compounds was lower in the albino-induced yellow leaves than in the normal green leaves (Figure 4). 

Although a genetic transformation system has yet to be established for tea, in the last decade, most identified genes involved in the formation of tea aroma compounds have been expressed using *Escherichia coli*, yeast, and insect cells, or in transient overexpression model plant systems and functionally characterized in vitro [5]. β-Glycosidases, including CsPD, CsGlus, and CsGTs, involved in the transformation of glycoside-bound aromas and free aromas in tea leaves have been intensively studied and functionally characterized [18,19,20,21,22]. Furthermore, several enzymes and genes involved in the final steps of the biosynthesis of tea aroma compounds, such as CsTPSs for (*S*)-linalool and (*E*)-nerolidol formation, and CsTSA and CsTSBs for indole formation, have been isolated, identified, and functionally characterized [23,24,25,26]. Some enzymes and genes involved in upstream pathways responsible for the formation of tea aroma compounds, such as CsLOXs for jasmine lactone formation and CsAAATs for benzaldehyde, benzyl alcohol, and methyl benzoate formation, have also been functionally characterized [27,28]. In the present study, the levels of genes involved in tea aroma formation, including *CsPD*, *CsGT1*, *GsGT2*, *CsLIS1*, *CsLIS2*, *CsLIS/NES-1*, and *CsLIS/NES-2*, were investigated to compare differences in the tea aroma biosynthetic abilities of albino-induced yellow leaves and normal green tea leaves (Figure 4E and Figure 5A). As the functions of most genes involved in tea aroma formation investigated in the present study have been validated, the differences in metabolic flux between albino-induced yellow leaves and normal green tea leaves were more reliable. These investigated genes showed no significant difference between albino-induced yellow leaves and normal green leaves. However, the content of some key precursors of aroma compounds, such as GDP, was lower in albino-induced yellow leaves (Figure 5B). 

## 4. Materials and Methods

### 4.1. Chemicals and Regents

*n*-Alkanes (C8–C40) were purchased from J&K Scientific, Beijing, China. Benzaldehyde, benzyl alcohol, (*Z*)-3-hexenyl acetate, methyl salicylate, and 2-phenylethanol were purchased from Wako Pure Chemical Industries Ltd, Osaka, Japan. Ethyl *n*-decanoate, geraniol, 1-hexanol, (*Z*)-3-hexenol, phenylacetaldehyde, α-farnesene, (*E*)-nerolidol, linalool, linalool oxide, polyvinylpolypyrrolidone (PVPP), and XAD-2 were purchased from Sigma-Aldrich Company Ltd., Louis, MO, USA. Geraniol was purchased from TCI Development Co., Ltd, Shanghai, China. The Quick RNA isolation kit was purchased from Huayueyang Biotechnology Co., Ltd., Beijing, China. The 2× SYBR Green Universal PCR Mastermix was purchased from Bio-Rad Laboratories, Richmond, CA, USA.

### 4.2. Plant Materials

Three or four leaves of *C. sinensis* cv. Yinghong No. 9 and its yellow mutant (a light-sensitive variant) were used in the present study. *C. sinensis* cv. Yinghong No. 9 yellow mutant (a light-sensitive variant) and its original cultivar were picked at the Tea Research Institute, Guangdong Academy of Agricultural Sciences (Yingde, Guangdong, China), in April, August, and December.

### 4.3. Extraction and Analysis of Endogenous Aroma Compounds in Tea Leaves

The method used for extraction and analysis of endogenous aroma compounds in tea leaves was taken from our previous study with some modifications [29]. Finely powdered sample (1 g, fresh weight) was extracted with dichloromethane (2.7 mL) containing ethyl decanoate (5 nmol) as an internal standardusing a shakerat room temperature overnight. The extraction solution was collected, dried using anhydrous sodium sulfate, and concentrated to 200 μL under a stream of nitrogen. The extract (1 μL) was then subjected to GC-MS analysis conforming on a GC-MS QP2010 SE (Shimadzu Corporation, Kyoto, Japan) equipped with GC-MS Solution software (Version 2.72, Shimadzu Corporation, Kyoto, Japan). Samples were injected into the GC injection port held at 230 °C for 1 min, with all injections made in splitless mode. Aroma compounds were separated on a SUPELCOWAX 10 column (30 m × 0.25 mm × 0.25 μm, Supelco Inc., Bellefonte, PA, USA). Helium was used as the carrier gas, with a velocity of 1 mL/min. The initial GC oven temperature was 60 °C for 3 min, which was ramped up to 240 °C at a rate of 4 °C/min, and then held at 240 °C for 20 min. Mass spectrometry (Shimadzu Corporation, Kyoto, Japan) was operated in full scan mode (mass range, *m*/*z* 40–200). 

The identification and quantitative analysis of the aroma compounds is summarized in Table S1 (Supplementary Information). 1-Hexanol, (*Z*)-3-hexenol, (*Z*)-3-hexenyl acetate, linalool, (*E*)-nerolidol, geraniol, α-farnesene, linalool oxide, methyl salicylate, phenylacetaldehyde, benzaldehyde, benzyl alcohol, and 2-phenylethanol were identified by direct comparison with authentic standards. The quantitative analyses of these compounds were based on calibration curves, which were constructed by plotting the concentration of each compound against the peak area of the authentic standard. Some aroma compounds, for which no authentic standards were available in our lab were identified by comparison with retention indices (RI). The RIs of the compounds were calculated from an *n*-alkane series (C8–C40) [30]. Compounds with minor differences (less than 20) between the experimental RI and RI values cited in the literature were confirmed as common components. The relative content of each compound was calculated by comparison with the peak area of ethyl *n*-decanoate (internal standard). Some aroma compounds for which no standards and reference RI values were available, were identified by comparison with mass spectra, and these compounds were tentatively identified. In addition, the quantitative analysis of these compounds was the same as the one identified based on the reference RI value. 

### 4.4. Extraction and Analysis of GBVs in Tea Leaves

The methods used for extraction and analysis of GBVs in tea leaves were according to the former study [22]. Five hundred mg (fresh weight) of finely powdered samples was extracted with 2 mL pre-cooling methanol by vortexing for 2 min followed by ultrasonic extraction in ice-cold water for 10 min. The extracts were purified using 2 mL cold chloroform and 0.8 mL cold water. The resulting upper layer was dried, and redissolved in 1 mL water. The resulting solution was mixed with 30 mg PVPP, stood for 60 min, and centrifuged (10,000×*g*, 4 °C, 10 min). The final supernatant was loaded to an Amberlite XAD-2 column (1 mL) and eluted with 5 mL water (to remove sugars etc.), 5 mL pentane:dichloromethane (2/1) (to remove free aroma compounds), and 5 mL methanol. The methanol eluent was dried using nitrogen stream, and redissolved in 800 μL of 50 mM citric acid buffer (pH 6.0) containing PD and β-glucosidase, and reacted at 37 °C for 16 h. Afterwards, 144 mg of sodium chloride was added to the reaction solution, and stood for 15 min followed by adding 0.5 nmol of ethyl decanoate as an internal standard. The solution was extracted by 800 μL extraction mixture (hexane:ethyl acetate, 1/1). After centrifugation, the extraction solution was collected, dried using anhydrous sodium sulfate, concentrated to 100 μL under a stream of nitrogen, and followed by GC-MS analysis. The GC-MS condition and quantitative analysis of aroma compounds from glycosidic hydrolysis were the same as those described above. 

### 4.5. Transcript Expression Analysis of Key Genes Involved in Formation of Tea Aroma Compounds

Total RNA was obtained using Quick RNA isolation Kit (Huayueyang Biotechnology Co., Ltd., Beijing, China). The cDNA was reversely transcribed from total RNA using PrimeScript RT Reagent Kit with gDNA Eraser (Takara Bio Inc., Kyoto, Japan) according to the manufacturer’s instruction. Gene transcript expression was determined by quantitative real time PCR (qRT-PCR). The reaction was performed in a 0.2 mL microtube containing iTaq^TM^ Universal SYBR^®^ Green Supermix (10 μL) (Bio-Rad, Hercules, CA, USA), 0.2 μM of each specific primer, 20-fold diluted cDNA (2 μL), and ddH_2_O (6 μL). The encoding elongation factor1 (*CsEF1*) was used as an internal reference gene. The related genes specific primers of qRT-PCR are shown in Table 1. The qRT-PCR was carried out on Roche LightCycle 480 (Roche Applied Science, Mannheim, Germany) under condition of one cycle of 95 °C for 60 s, 40 cycles of 95 °C for 15 s, and 60 °C for 30 s. A melt curve was performed at the end of each reaction to verify PCR product specificity. The 2^−∆∆ct^ method was used to calculate the relative expression level. Changes in mRNA levels of related genes were normalized to that of *CsEF1*. 

### 4.6. Extraction and Analysis of GDP in Tea Leaves

The method used was taken from our previous studies with some modifications [23,29]. Finely powdered sample (200 mg, fresh weight) was extracted with buffer (2 mL; 50 mM Tris/HCl, pH 8.0, 20 mM DTT, 20 mM MgCl_2_, and 5% glycerin) containing 10 mM sodium molybdate as phosphatase inhibitor by vortexing for 2 min, followed by ultrasonic extraction in ice-cold water for 10 min, and centrifugation (10,000×*g*, 4 °C, 10 min). The supernatant (1.6 mL) was extracted with hexane (1.6 mL) five times to remove free aromas. The above buffer (800 μL) was added and the aqueous fraction was incubated at 37 °C for 30 min with 1 M H_2_SO_4_ (2 mL). The reaction solution was cooled down for 3 min on ice and then the reaction was stopped by adding 100 μL of 4 M NaOH. The resultant mixture was extracted with 1 mL of hexane containing 0.5 nmol of ethyl decanoate as the internal standard. The extract was then subjected to GC-MS analysis as described above. The GDP content was calculated based on standard curves which were constructed by plotting the concentration of linalool against the peak area of the authentic standard.

### 4.7. Statistical Analysis

Statistical analysis was performed using SPSS software, version 18.0 (SPSS Inc., Chicago, IL, USA). Two-tailed student’s *t* test was used to determine the differences between the normal and albino tea leaves.

## 5. Conclusions

Herein, we detected the differential accumulation of aroma metabolites (aroma compounds) in albino-induced yellow tea leaves and normal green leaves. The albino-induced yellow tea leaves had lower amounts of free tea aroma compounds than the normal green tea leaves. In addition, most glycosidically bound aroma compounds in albino-induced yellow leaves were lower than those in normal green leaves. The lower content of linalool in albino-induced yellow tea leaves was due to lower GDP content compared with normal green tea leaves. This information will help us realize profiles of tea aromas and their formation in albino tea leaves. Furthermore, the results obtained in this study will help us to precisely evaluate the advantages (high amino acids) and disadvantages (low aromas) of albino-induced yellow tea leaves used to make tea products.

## Figures and Tables

**Table 1:** The primers used for quantitative real time PCR (qRT-PCR) in the study.

Gene	Accession Number	Forward Primer 5′-3′	Reverse Primer 5′-3′
*CsEF1*	KA280301.1	TTGGACAAGCTCAAGGCTGAACG	ATGGCCAGGAGCATCAATGACAGT
*CsPD*	AB088027.1	CCAAAGGTTCGGAATTGTCTATG	GCGCTTTTAGTCATACACCGA
*CsGT1*	AB847092	TGAAGAAGGAAGCAGAAGAAGC	GGCTCATGATTCAACCGG
*CsGT2*	AB847093	GAGGACATAAGGATTAAAGCGAG	TTTTCAACCCACTTAAATATTTCAATA
*CsLIS1*	KF006849	ACATTGCAAGGATGGTTCC	ATGAGCATTACAGGTGCTAGCT
*CsLIS2*	KY033151	GTCAATGTTCCGTGATACTGTTTC	ACACCAAGATAGACACCCTACTTTC
*CsLIS/NES-1*	KF006849	TCCAACCCCTCAATACAGAAAGACTATC	TTGGCTTTGTAGAAGTGCTTCAATCTC
*CsLIS/NES-2*	-	GAATGACAATCCAGGCATTG	TGGTGAGAATGGATTTGGAG

*EF1*, *encoding elongation factor 1*; *PD*, *β-primeverosidase*; *GT*, *glycosyltransferase*; *LIS*, *linalool synthase*; *LIS/NES*, *linalool synthase/(E)-nerolidolsynthase*.

## Supplementary Materials

The following are available online, Figure S1: GC-MS TIC chromatograms of endogenous free aroma compounds in normal green and albino-induced yellow tea leaves collected in April, August, and December, respectively; Table S1: Identified aroma compounds in the study.

## Abbreviations

The following abbreviations are used in this manuscript:

VFADs	Aroma fatty acid derivatives
VTs	Aroma terpenes
VPBs	Aroma phenylpropanoids/benzenoids
PVPP	Polyvinylpolypyrrolidone
GDP	Geranyl diphosphate
RI	Retention indices
GBVs	Glycosidically bound aromas
EF1	Encoding elongation factor 1
PD	β-Primeverosidase
GT	Glycosyltransferase
LIS	Linalool synthase
LIS/NES	Linalool synthase/(*E*)-nerolidol synthase
