# Supplementary material for: Differential Accumulation of Aroma Compounds in Normal Green and Albino-Induced Yellow Tea (Camellia sinensis) Leaves
Source: Molecules. 2018 Oct 18;23(10):2677. doi: 10.3390/molecules23102677 (PMC6222872; doi:10.3390/molecules23102677)
Supplement: Supplementary file 1 [file molecules-23-02677-s001.pdf]

molecules

Article

Differential Accumulation of Aroma Compounds in Normal Green and Albino-Induced Yellow Tea (*Camellia sinensis*) Leaves

Fang Dong [1,†], Lanting Zeng [2,3,†], Zhenming Yu [2,3], Jianlong Li [4], Jinchi Tang [4], Xinguo Su [1,*], and Ziyin Yang [2,3,*]

1 Guangdong Food and Drug Vocational College, Longdongbei Road 321, Tianhe District, Guangzhou 510520, China; dongfangxyz@163.com
2 Key Laboratory of South China Agricultural Plant Molecular Analysis and Genetic Improvement & Guangdong Provincial Key Laboratory of Applied Botany, South China Botanical Garden, Chinese Academy of Sciences, Xingke Road 723, Tianhe District, Guangzhou 510650, China; zenglanting@scbg.ac.cn (L.Z.) ; zhenming311@scbg.ac.cn (Z.Y.)
3 College of Advanced Agricultural Sciences, University of Chinese Academy of Sciences, No.19A Yuquan Road, Beijing 100049, China
4 Tea Research Institute, Guangdong Academy of Agricultural Sciences & Guangdong Provincial Key Laboratory of Tea Plant Resources Innovation and Utilization, Dafeng Road 6, Tianhe District, Guangzhou 510640, China; skylong.41@163.com (J.L.); tangjinchi@126.com (J.T.)

* Correspondence: suxg@gdyzy.edu.cn (X.S.); zyyang@scbg.ac.cn (Z.Y.); Tel.: +86-20-2885-4755 (X.S.); +86-20-3807-2989 (Z.Y.); Fax: +86-20-3807-2989 (Z.Y.)

† These authors contributed equally to this work.

molecules

MDPI

Supplemantary information

Supplementary figure

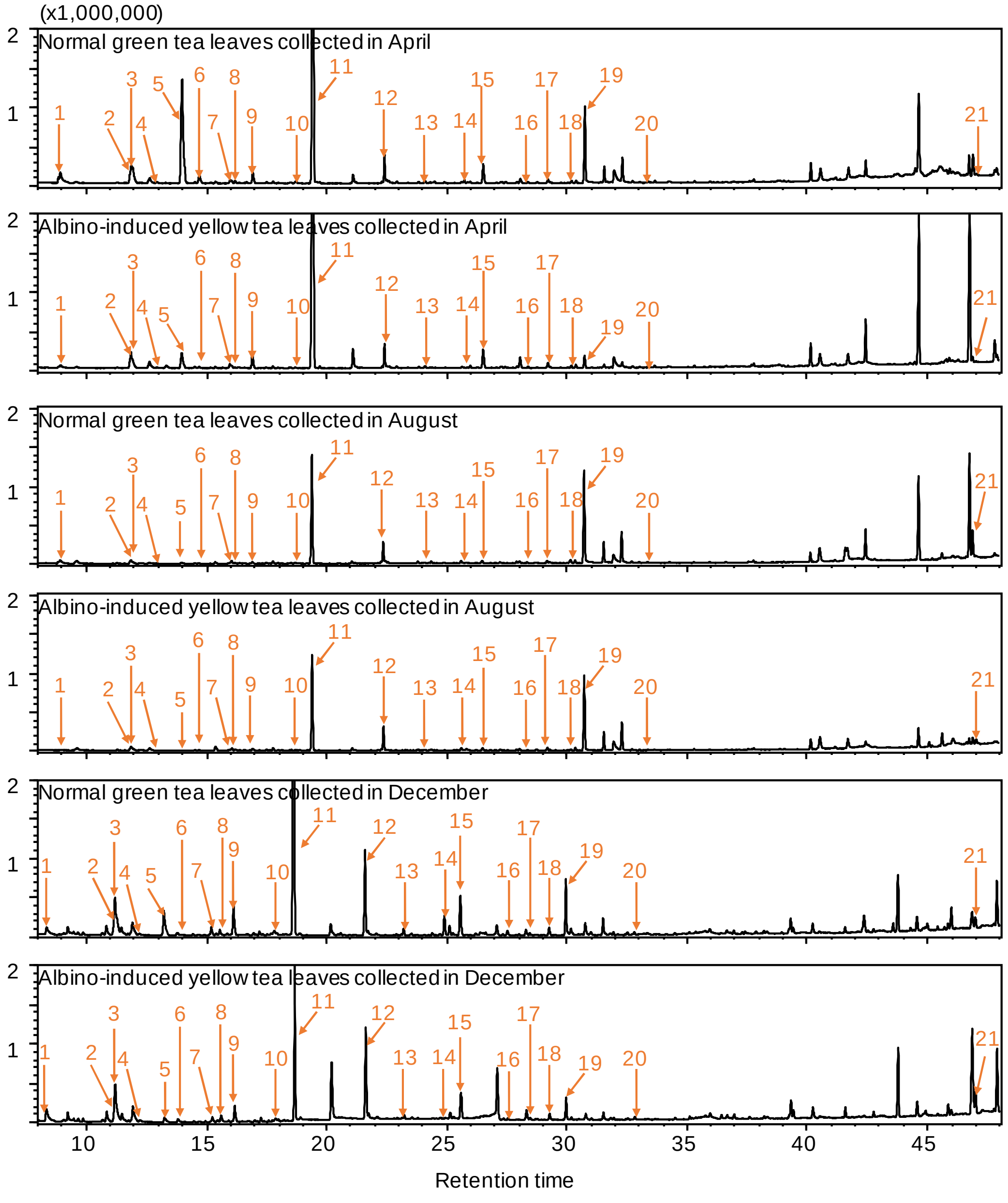

Figure S1 GC-MS TIC chromatograms of endogenous free aroma compounds in normal green and albino-induced yellow tea leaves collected in April, August, and December, respectively 1), 2-Hexenal; 2), 2-Heptanol; 3), (*Z*)-3-Hexenyl acetate; 4), 1-Hexanol; 5), (*Z*)-3-Hexenol; 6), (*E*)-2-Hexenol; 7), Linalool oxide I; 8), 3-Octenol; 9), Linalool oxide II; 10), Benzaldehyde; 11), Linalool; 12), Phenylacetaldehyde; 13), *α*-Terpineol; 14), *α*-Farnesene; 15), Methyl salicylate; 16), Geraniol; 17), Benzyl alcohol; 18), 2-Phenylethanol; 19), Phytol, acetate; 20), (*E*)-Nerolidol; 21), Phytol. The tea samples collected in April and August were simultaneously analyzed by GC-MS, while the tea samples collected in December were solely analyzed by GC-MS. Therefore, the GC-MS retention times were a little different.

Supplementary tables

Table S1 Identified aroma compounds in the study.

No.	Aroma compound	RI[a]	RI[b]	Identification[c]	Quantitative analysis[d]
1	2-Hexenal	1219	1225[1]	RI, MS	Internal Std
2	2-Heptanol	1314	1332[2]	RI, MS	Internal Std
3	(Z)-3-Hexenyl acetate	1317	-	RI, MS, Std	Std
4	1-Hexanol	1347	1351[3]	RI, MS, Std	Std
5	(*Z*)-3-Hexenol	1379	1395[1], 1387[4]	RI, MS, Std	Std
6	(*E*)-2-Hexenol	1401	1418[1]	RI, MS	Internal Std
7	Linalool oxide I	1439	1455[1], 1448[5]	RI, MS, Std	Std
8	3-Octenol	1446	1444[6]	RI, MS	Internal Std
9	Linalool oxide II	1468	1483[1]	RI, MS, Std	Std
10	Benzaldehyde	1523	1528[1], 1540[5]	RI, MS, Std	Std
11	Linalool	1544	1555[1], 1542[6]	RI, MS, Std	Std
12	Phenylacetaldehyde	1659	1647[1], 1646[4]	RI, MS, Std	Std
13	α-Terpineol	1718	1708[1]	RI, MS	Internal Std
14	α-Farnesene	1761	1754[1]	RI, MS, Std	Std
15	Methyl salicylate	1781	1780[1]	RI, MS, Std	Std
16	Geraniol	1842	1861[1]	RI, MS, Std	Std
27	Benzyl alcohol	1870	1887[1], 1874[2]	RI, MS, Std	Std
28	2-Phenylethanol	1905	1924[1]	RI, MS, Std	Std
19	Phytol, acetate	1926	-	MS	Internal Std
20	(*E*)-Nerolidol	2036	2050[1], 2040[7]	RI, MS, Std	Std
21	Phytol	2607	2624[1]	RI, MS	Internal Std

[a] The retention indices (RI) values were calculated from *n*-alkanes series (C8-C40).

[b] The RI values were the reference values from the literatures.

[1] Jeon et al., 2017; DB-Wax column (60 m× 0.25 mm× 0.25 μm). [2] Lee & Shibamoto, 2001; DB-Wax column (30 m×0.25 mm×0.25 μm). [3] Elmore et al., 2004; SupelcoWax-10 column (60 m×0.25 mm×0.25 μm). [4] Kumazawa & Masuda, 2002; DB-Wax column (30 m× 0.25 mm× 0.25 μm). [5] Soria, Martínez-Castro, & Sanz, 2008; SupelcoWax-10 column (50 m×0.25 mm×0.25 μm). [6] Cavaleiro, Salgueiro, Miguel, & da Cunha, 2004; SupelcoWax-10 column (30 m×0.2 mm×0.2 μm). [7] Choi, Kim, & Sawamura, 2002; DB-Wax column (60 m×0.25 mm×0.25 μm). -, lacking of reference RI value from literatures.

[c] Methods of identification. RI, identification based on retention index; MS, Identification based on comparison of mass spectra; Std, Identification based on authentic standard. When only MS is available for the identification of a compound, it must be considered as a tentative identification.

[d] Methods of quantitative analysis. Std, quantitative analysis based on authentic standard. Internal Std, quantitative analysis based on ethyl *n*-decanoate (internal standard).

molecules

MDPI

Supplementary references

1. Jeon, D.B.; Hong, Y.S.; Lee, G.H.; Park, Y.M.; Lee, C.M.; Nho, E.Y.; Jamila, N.; Khan, N.; Kim, K.S. Determination of volatile organic compounds, catechins, caffeine and theanine in Jukro tea at three growth stages by chromatographic and spectrometric methods. *Food Chem.* **2017**, *219*, 443–452.
2. Lee, K.G.; Shibamoto, T. Antioxidant property of aroma extract isolated from clove buds [*Syzygium aromaticum* (L.) Merr. et Perry]. *Food Chem.* **2001**, *74*, 443–448.
3. Elmore, J.S.; Warren, H.E.; Mottram, D.S.; Scollan, N.D.; Enser, M.; Richardson, R.I.; Wood, J.D. A comparison of the aroma volatiles and fatty acid compositions of grilled beef muscle from Aberdeen Angus and Holstein-Friesian steers fed diets based on silage or concentrates. *Meat Science* **2004**, *68*, 27–33.
4. Kumazawa, K.; Masuda, H. Identification of potent odorants in different green tea varieties using flavor dilution technique. *J. Agr. Food Chem.* **2002**, *50*, 5660−5663.
5. Soria, A.C.; Martínez-Castro, I.; Sanz, J. Some aspects of dynamic headspace analysis of volatile components in honey. *Food Res. Int.* **2008**, *41*, 838–848.
6. Cavaleiro, C.; Salgueiro, L.R.; Miguel, M.G.; da Cunha, A.P. Analysis by gas chromatography–mass spectrometry of the volatile components of *Teucrium lusitanicum* and *Teucrium algarbiensis*. *J. Chromatogr. A* **2004**, *1033*, 187–190.
7. Choi, H.S.; Kim, M.S.L.; Sawamura, M. Constituents of the essential oil of cnidium officinale Makino, a Korean medicinal plant. *Flavour Frag. J.* **2002**, *17*, 49–53.
